# Comparative Accuracy of Semiconductor Single-Photon Emission Computed Tomography (SPECT) Versus Cardiac Magnetic Resonance (CMR) for Myocardial Viability Assessment

**DOI:** 10.7759/cureus.83679

**Published:** 2025-05-07

**Authors:** Arata Sano, Satoaki Matoba, Koshi Miyake, Sachiyo Ono, Takeshi Tada, Takeshi Maruo, Kazushige Kadota

**Affiliations:** 1 Department of Cardiovascular Medicine, Kyoto Prefectural University of Medicine, Kyoto, JPN; 2 Department of Cardiovascular Medicine, Kurashiki Central Hospital, Kurashiki, JPN

**Keywords:** cardiac magnetic resonance (cmr), cardiac spect, ischemic cardiomyopathy, myocardial viability, omi

## Abstract

Background

Evaluating myocardial viability is critical for developing optimal strategies for ischemic cardiomyopathy. While semiconductor single-photon emission computed tomography (D-SPECT) demonstrated higher image quality than conventional Anger cameras for assessing myocardial viability, its accuracy compared to cardiac magnetic resonance (CMR) in the same patient cohort is still unknown.

Methods

We conducted a retrospective study on patients with known or suspected coronary artery disease who underwent D-SPECT and CMR within 100 days. Rest deficit score on D-SPECT and depth of contrast enhancement on late gadolinium enhancement (LGE) in CMR were classified and compared using a 16-segment analysis. Follow-up echocardiography was performed about a year after the initial examinations, detecting whether the optimal medical therapy (OMT) was followed by invasive therapy (coronary artery bypass grafting [CABG] or percutaneous coronary intervention [PCI]). The five-year survival rates were also compared.

Results

The study comprised 336 segments from 21 consecutive patients collected between January 2015 and December 2017. Using LGE as a viability criterion, a D-SPECT score of three had the highest diagnostic accuracy (area under the curve: 0.97). Follow-up echocardiography showed significant improvements in left ventricular ejection fraction in patients receiving OMT + PCI/CABG compared to OMT alone (OMT vs. OMT + PCI/CABG; 1.5% ± 3.4% vs. 7.1% ± 5.0%, p = 0.008); five-year survival rate did not significantly differ between the groups.

Conclusions

Deficits observed at rest on D-SPECT and LGE extent on CMR showed a strong correlation in evaluating myocardial viability, implying that D-SPECT is a viable alternative to CMR for this purpose.

## Introduction

The prevalence of heart failure (HF) has increased in recent years as a result of population aging and improved post-diagnosis survival rates [1]. The most common cause of heart failure with reduced ejection fraction is ischemic heart disease [2], contributing significantly to the rising global incidence of HF [3].

Ischemic cardiomyopathy is a type of HF that results from chronic left ventricular (LV) systolic dysfunction caused by underlying coronary artery disease (CAD) [4]. An important pathophysiological hallmark of ischemic cardiomyopathy is the coexistence of scar tissue with fibrosis replacement alongside areas of dysfunctional but viable myocardium [5]. Myocardial viability refers to myocardial segments that, despite being dysfunctional, have the potential for functional recovery upon reestablishing blood supply [5-8]. The presence of viable myocardium has significant implications for the treatment of patients with ischemic cardiomyopathy.

Various imaging techniques are used to assess myocardial viability. The previously used single-photon emission computed tomography (SPECT) has recently lost popularity due to the superior accuracy of late gadolinium enhancement (LGE) in cardiac magnetic resonance imaging (CMR) for detecting myocardial viability [9], with delayed contrast-enhanced MRI providing insights into the extent of infarction and myocardial viability [10].

Conversely, D-SPECT, semiconductor SPECT that uses cadmium-zinc-telluride (CZT) detectors and rotating detector columns, outperforms its conventional counterpart in terms of radiation exposure and scan time while maintaining image quality [11-14]. A meta-analysis found that 50% of LV wall hyperenhancement was a viable cutoff for determining LV segment viability (i.e., <50% hyperenhancement was deemed viable and >50% hyperenhancement was deemed nonviable) [15].

A recent multicenter clinical trial found that semiconductor SPECT correlates well with traditional SPECT for rest-stress myocardial perfusion and function [16]. However, studies comparing the diagnostic accuracy of semiconductor SPECT, specifically D-SPECT, for assessing myocardial viability to CMR are lacking, and the current work aims to fill this gap by verifying the accuracy of D-SPECT in evaluating myocardial viability in comparison to CMR.

## Materials and methods

Study cohort

**Figure 1:**
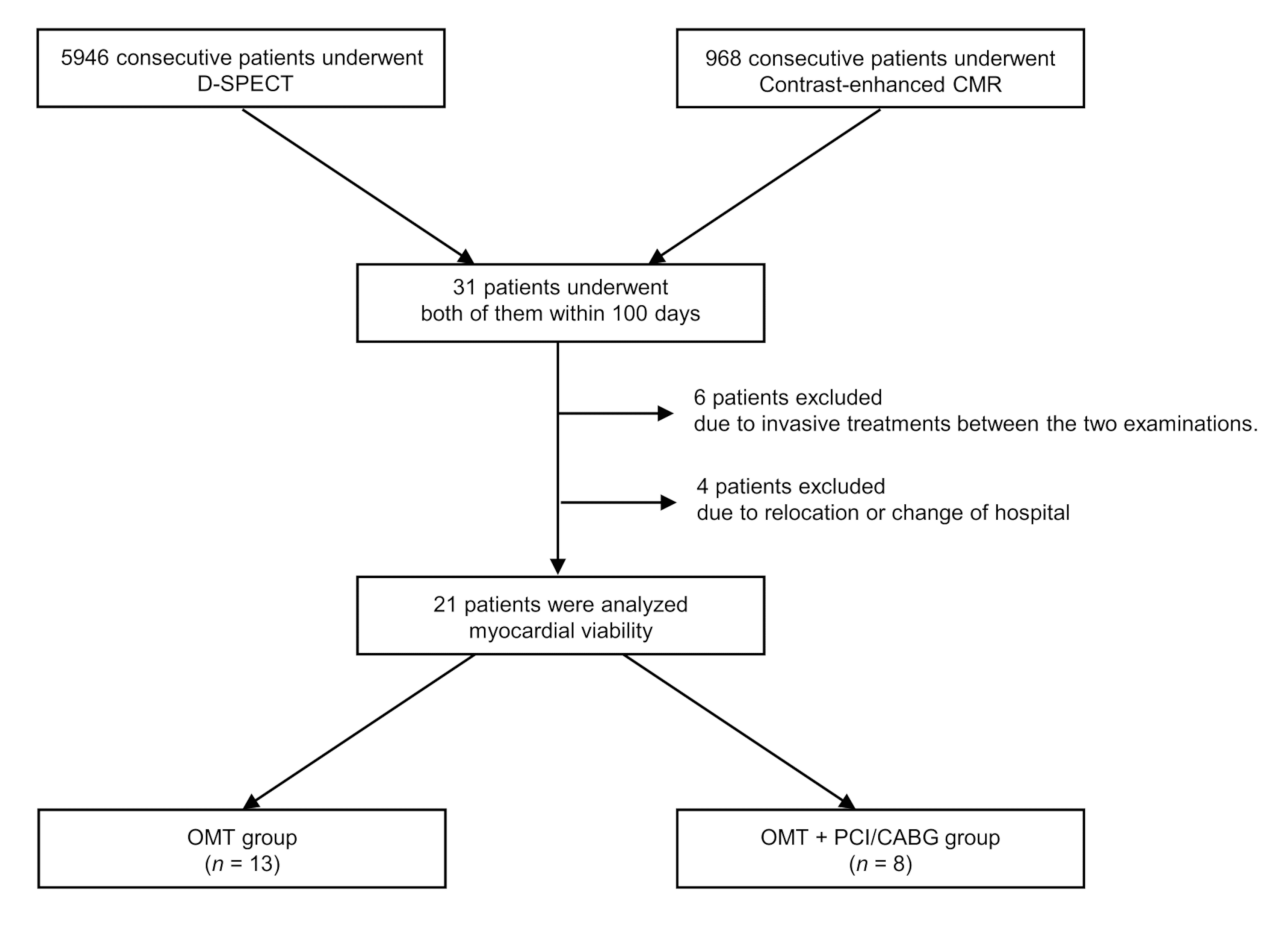
Study flowchart. From January 2015 to December 2017, 21 consecutive patients were enrolled in this study. Those who underwent invasive therapy (PCI or CABG) were excluded.

Between January 2015 and December 2017, Kurashiki Central Hospital performed D-SPECT imaging in 5,946 patients and CMR in 2,057 patients, with contrast-enhanced CMR performed in 968 cases. In cases where it was difficult to decide on a treatment plan using SPECT or CMR alone, both tests were performed. We enrolled 21 consecutive patients who received both D-SPECT and CMR for cardiac function evaluation within a 100-day interval (Figure 1). Patients who underwent PCI or CABG between the two imaging examinations were excluded to maintain the stability of myocardial viability status during evaluation. These patients also underwent transthoracic echocardiography. The patients were divided into two groups: the OMT group included those treated only with OMT, while the OMT + PCI/CABG group contained the patients treated with OMT plus PCI or CABG. Follow-up echocardiography was performed about a year after the initial examinations on all patients, and the five-year survival rate between the two groups was compared. This study was approved by the Ethics Committee of Kurashiki Central Hospital. All data were anonymized in accordance with institutional and ethical standards.

Cardiac MRI

Cardiac MRI was performed with Ingenia 1.5 T MR systems (Philips Medical Systems, Best, Netherlands) and gadobutrol (Gadovist: Bayer Schering Pharma, Berlin, Germany, or Magnescope: Fuji Pharma, Tokyo, Japan) as the contrast agent. Standard ECG-gated steady-state free precession pulse sequences were used to acquire images [17]. Delayed-enhancement images of myocardial viability were obtained ≈10 minutes after intravenous gadobutrol injection (Gadovist 0.1 ml/kg or Magnescope 0.3 ml/kg) using a segmented inversion recovery fast gradient echo sequence. The imaging parameters for the 10 min LGE scan were as follows: repetition time (TR) 4.2 ms; echo time (TE) 1.34 ms; flip angle (FA) 15°; readout bandwidth (BW) 298.6 Hz/px; matrix 256  ×  222; field of view (FOV) 400 mm; parallel imaging applied (Compressed SENSE); and slice thickness 8 mm. This sequence followed the same prescription as the short-axis cine sequence to ensure image registration. The areas of hyperenhancement (LGE) were traced manually and reported as a percentage of LV mass. The depth of contrast in LGE was classified to assess the cardiac viability (0%: 0%, 1%-25%: 25%, 26%-50%: 50%, 51%-75%: 75%, 76%-100%: 100%) [18].

D-SPECT

D-SPECT imaging (Spectrum Dynamics Medical, Caesarea, Israel) was conducted using a one-day stress/rest protocol with 99mTc-tetrofosmin or 201TlCl as the radiopharmaceutical. The stress protocol involved injecting adenosine at a rate of 120 µg/kg/min for 6 minutes, followed by an intravenous injection of 99mTc-tetrofosmin or 201TlCl, usually 3 minutes later. For 99mTc-tetrofosmin, stress MPI was performed 45 minutes after injection, followed by rest MPI 1 hour later. For 201TlCl, stress and rest MPI were performed 5-10 minutes and 3 hours after injection, respectively. The energy window was set at 140 keV ± 10% for 99mTc and 70 keV ± 15% for 201Tl. Other acquisition parameters were optimized for image quality and count statistics [11]. The acquired images were reconstructed and analyzed using dedicated software, with a 16-segment model and a 5-point model (0: normal uptake; 1: mildly reduced uptake; 2: moderately reduced uptake; 3: severely reduced uptake; 4: almost no uptake) based on American Society of Nuclear Cardiology (ASNC) guidelines [19]. The choice of radiopharmaceutical (99mTc-tetrofosmin or 201TlCl) was based on institutional availability and clinical discretion. All images were interpreted by experienced nuclear cardiologists and radiologists blinded to clinical data and outcomes.

Transthoracic echocardiography

Transthoracic echocardiography was performed following American Society of Echocardiography (ASE) guidelines, with each of the 16 segments’ wall motion score and depth diameter measured [20].

Statistical analysis

The sensitivity, specificity, and negative and positive predictive values were calculated for D-SPECT using the CMR cutoff for evaluating myocardial viability. Receiver operating characteristic (ROC) curves were used to assess the discriminative ability of D-SPECT, and the area under the curve was used to quantify overall performance in each segment of all the patients. Spearman’s rank correlation was used to assess the relationship between D-SPECT and CMR scores. To identify differences in survival between the two groups, the Kaplan-Meier curve was used to examine the five-year prognosis. Statistical significance was determined as a p-value of <0.05. This study represents a retrospective convenience sample based on the clinical availability of both imaging modalities. No formal power or sample size calculation was performed. The limited sample size is acknowledged as a constraint, particularly for comparisons involving long-term outcomes, because each patient contributed 16 myocardial segments, and potential intra-patient correlation was considered in the interpretation of segment-level analyses, although formal mixed-effects modeling was not performed.

## Results

Twenty-one consecutive patients (16 men and five women, average age 62.3 years) underwent D-SPECT and CMR (Table 1). No patients experienced any cardiovascular events or underwent invasive treatments between the two examinations. Each patient’s diagnostic images were successfully obtained using both methods. The measurement data of LV in each patient were all patients underwent follow-up transthoracic echocardiography around a year later following D-SPECT and CMR. We compared the two echocardiographic studies to determine whether the patient’s cardiac function, whose cardiac viability was maintained, had recovered or not. Eight patients had invasive therapy (PCI was performed for three patients, and CABG was performed for five patients). The remaining 13 patients underwent OMT with beta-blockers and renin-angiotensin system (RAS) inhibitors. Viability findings obtained from D-SPECT and CMR were considered in clinical decision-making regarding revascularization. We divided the patients into two groups: the OMT group and the OMT + PCI/CABG group. The percentage of diabetic patients was significantly higher in the OMT + PCI/CABG group, but there were no significant differences in other patient characteristics between the two groups (Table 2). LV data showed no significant difference between the two groups (Table 3). Medications such as beta-blockers and RAS inhibitors were introduced at a high rate at discharge in both groups with no significant differences. At baseline, there was no significant difference in left ventricular ejection fraction (LVEF) between the OMT group and the OMT + PCI/CABG group (OMT group vs. OMT + PCI/CABG group; 42.4 ± 12.5% vs. 35.7 ± 13.0%, p = 0.36).

**Table 1:** Patient Baseline Characteristics

Parameter	Value
Number of patients	21
Male (%)	76%
Age (years)	62.3 ± 14.2
Height (cm)	165.0 ± 5.7
Weight (kg)	68.0 ± 12.9
Body Surface Area (m²)	1.74 ± 0.18
Hypertension (%)	76%
Dyslipidemia (%)	71%
Diabetes (%)	29%
Familial History (%)	5%
Smoking (%)	76%
Post MI (%)	86%
Post PCI/CABG (%)	57%

**Table 2:** LV Measurement Data from CMR

Measurement	Value
LVEDV (ml)	200.3 ± 83.5
LVESV (ml)	137.1 ± 84.0
LVEF (%)	36.1 ± 13.7
LV mass (g)	147.2 ± 32.4
LV mass index (g/m²)	84.6 ± 17.2
LVDd (mm)	58.0 ± 8.6
LVDs (mm)	45.1 ± 12.4
IVST (mm)	12.1 ± 2.2
PWT (mm)	8.8 ± 1.8

**Figure 2:**
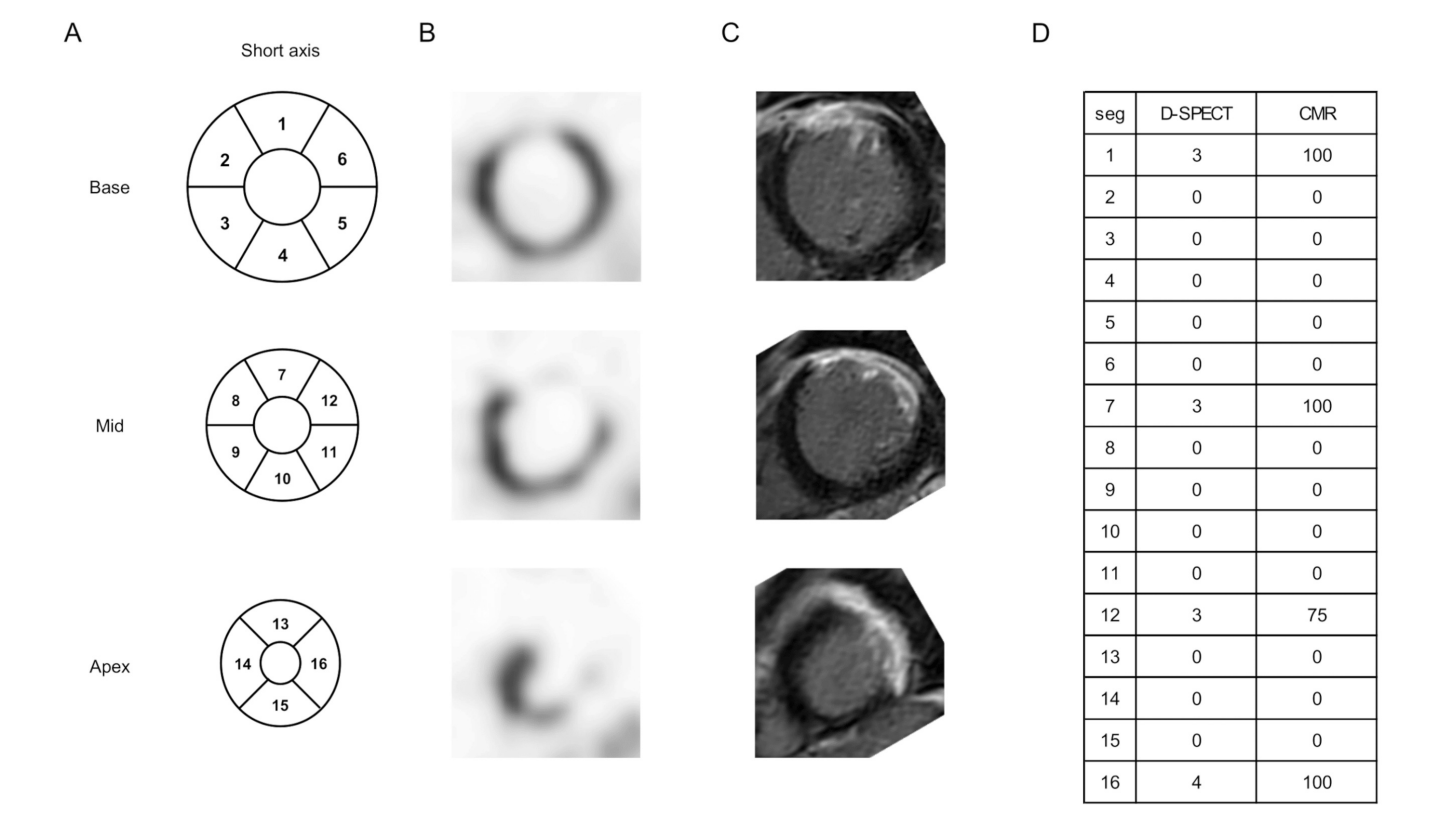
Representative case with D-SPECT and CMR images. (A) Scheme of 16 segments of left ventricular. (B) D-SPECT image. (C) CMR image. (D) Scores of D-SPECT and CMR in each segment.

A representative case is a 43-year-old male admitted with congestive HF (Figure 2). HFmrEF (LVEF: 42%) was discovered, and because myocardial viability was determined to have vanished, he was treated with optimal medical therapy (OMT). LVEF improved to 49% at 1-year Follow-up echocardiography, and the patient had no cardiac events for the next 5 years.

**Figure 3:**
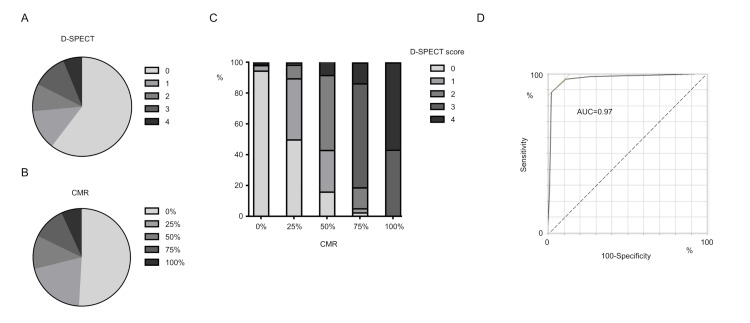
Distribution of D-SPECT and CMR scores. (A) D-SPECT score distribution. (B) CMR score distribution. (C) Correlation between D-SPECT and CMR. (D) Receiver operating characteristic for detecting myocardial viability.

While examining the 336 collected myocardial segments, we discovered a link between the depth of contrast in LGE on CMR and the deficit score at rest on D-SPECT (Figure 3a, 3b). D-SPECT scores increased with greater depths of LGE on CMR, indicating a significant relationship between the two methods (Figure 3c). Using a well-known CMR cutoff of 75% loss of myocardial viability [15], we compared the detection accuracy of D-SPECT and CMR. The area under the curve (AUC) was 0.97, with a sensitivity of 87.5%, specificity of 97.8%, positive predictive value of 90.9%, negative predictive value of 96.9%, and an overall accuracy of 95.8% (Fig. 3d). Moreover, a strong positive correlation between the two modalities was observed using Spearman’s rank correlation coefficient (ρ = 0.81, p < 0.001).

All patients underwent Follow-up transthoracic echocardiography around a year later following D-SPECT and CMR. We compared the two echocardiographic studies to determine whether the patient’s cardiac function whose cardiac viability was maintained had recovered or not. 8 patients had invasive therapy (PCI was performed for three patients, and CABG was performed for five patients). The remaining 13 patients underwent OMT with beta-blockers and renin-angiotensin system (RAS) inhibitors. We divided the patients into two groups: the OMT group and the OMT + PCI/CABG group. The percentage of diabetic patients was significantly higher in the OMT + PCI/CABG group, but there were no significant differences in other patient characteristics between the two groups (Table 3). LV data showed no significant difference between the two groups (Table 4). Medications such as beta-blockers and RAS inhibitors were introduced at a high rate at discharge in both groups with no significant differences (Table 5).

**Table 3:** Patient characteristics: OMT vs. OMT + PCI/CABG Post MI: Post-myocardial infarction, PCI: Percutaneous coronary intervention, CABG: Coronary artery bypass grafting, OMT: Optimal medical therapy, BSA: Body surface area

	OMT (n=13)	OMT + PCI/CABG (n=8)	p-value
Male (%)	11 (85)	5 (63)	0.25
Age	64.6	58.6	0.37
Height (cm)	165.9	163.5	0.44
Weight (kg)	69.4	65.9	0.57
BSA (m^2^)	1.77	1.71	0.48
Hypertension (%)	9 (69)	7 (88)	0.34
Dyslipidemia (%)	8 (62)	5 (63)	0.96
Diabetes (%)	0 (0)	6 (75)	<0.001
Familial history (%)	0 (0)	1 (13)	0.19
Smoking (%)	11 (85)	5 (63)	0.25
Post MI (%)	10 (77)	8 (100)	0.14
Post PCI/CABG (%)	8 (62)	4 (50)	0.60

**Table 4:** LV Measurement data: OMT vs. OMT + PCI/CABG LVDEV: Left ventricular diastolic volume, LVESV: Left ventricular end-systolic volume, LVEF: Left ventricular ejection fraction, LV: Left ventricle, LvDd: Left ventricular diastolic diameter, LVD: Left ventricular diameter, IVST: Interventricular septal thickness, PWT: Posterior wall thickness, OMT: Optimal medical therapy, PCI: Percutaneous coronary intervention, CABG: Coronary artery bypass grafting

	OMT (n=13)	OMT + PCI/CABG (n=8)	p-value
LVEDV (ml)	198.4	203.5	0.90
LVESV (ml)	131.2	146.8	0.70
LVEF (%)	39.5	30.5	0.16
LV mass (g)	145.3	150.3	0.75
LV mass index (g/m^2^)	82.0	88.6	0.42
LVDd (mm)	57.0	59.5	0.55
LVDs (mm)	42.0	50.1	0.16
IVST (mm)	12.6	11.2	0.18
PWT (mm)	9.4	8.0	0.10

**Table 5:** Medication usage at discharge RAS: Renin-angiotensin system, OMT: Optimal medical therapy, CABG: Coronary artery bypass grafting, PCI: Percutaneous coronary intervention

	OMT(n=13)	OMT + PCI/CABG (n=8)	p-value
β-blocker (%)	12 (92)	7 (88)	0.72
RAS inhibitor (%)	8 (62)	5 (63)	0.96
Ca-blocker (%)	9 (69)	7 (88)	0.34
Statin (%)	10 (77)	5 (63)	0.48

Follow-up echocardiography comparison showed that patients who received invasive therapy (PCI or CABG) along with OMT had significantly higher LVEF compared to those who only received OMT (OMT group vs. OMT + PCI/CABG group; 1.46 ± 3.37% vs. 7.13 ± 5.01%, p = 0.008) (Figure 2). To identify differences in survival between the two groups, the Kaplan-Meier curve was used to examine the five-year prognosis. However, the five-year survival rate did not differ significantly between the two groups (OMT group vs. OMT + PCI/CABG group; 83.3 % vs. 85.7%) (Figure 2).

**Figure 4:**
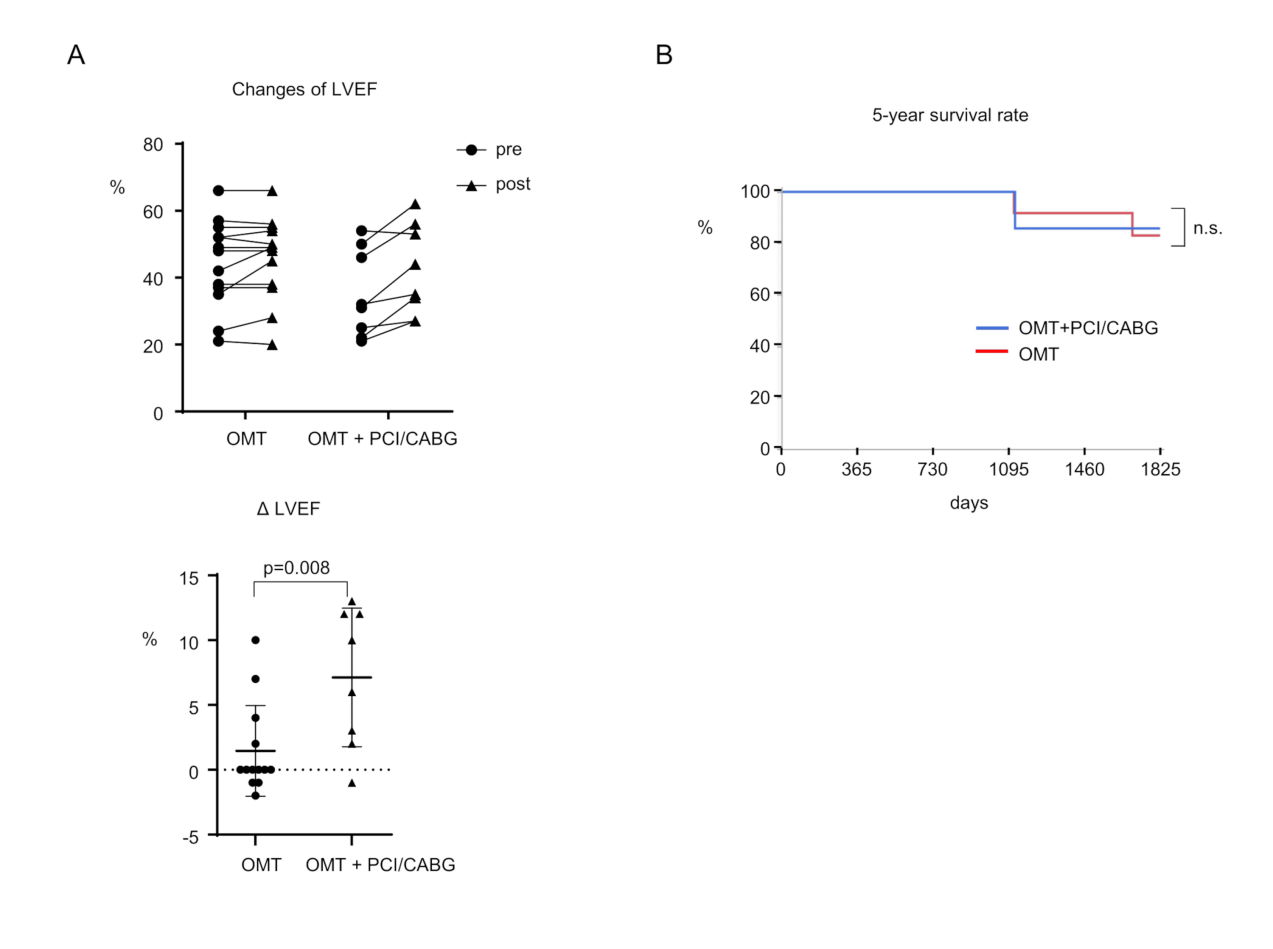
Follow-up data after the examinations. (A) Change in LVEF of the patients after one year between the two groups. (B) Kaplan–Meier curve of the five-year survival rate between the two groups. LVEF: Left ventricular ejection fraction

## Discussion

Our study presents one of the first direct comparisons of the diagnostic accuracy of D-SPECT and CMR for assessing myocardial viability in patients with known or suspected coronary artery disease. Our findings shed light on several important issues, including the comparative efficacy of D-SPECT and CMR, the implications for clinical decision-making, and potential directions for future research.

First and foremost, our findings reaffirm the diagnostic capabilities of D-SPECT in assessing myocardial viability, with a high degree of correlation with CMR findings. This validation of D-SPECT as a reliable alternative to CMR has significant clinical implications, especially in situations where CMR may be contraindicated or unavailable. For example, patients with renal impairment or who are unable to tolerate the confines of an MRI machine may benefit greatly from the availability of D-SPECT as a viable imaging modality. Furthermore, D-SPECT’s shorter scan time and lower radiation exposure make it a more practical tool for assessing myocardial viability. The most effective SPECT protocols, as previously reported, for evaluating myocardial viability -either the late redistribution (18-24 hours) imaging or the 4-hour redistribution image followed by a small reinjection of 201Tl -have been time-consuming [21]. D-SPECT overcomes these timing challenges, providing a quicker and more efficient imaging solution. Similarly, although 18FDG-PET is utilized for detecting cardiac viability [22], it requires more time compared to D-SPECT and presents limitations for diabetic patients due to the demanding metabolic preparation required [23].

Second, our findings emphasize the importance of individualized treatment strategies based on myocardial viability assessment. There were significant improvements in LVEF in patients who underwent invasive treatments, such as PCI or CABG, after confirmation of myocardial viability by both D-SPECT and CMR. Although no formal subgroup analysis was performed, this highlights the importance of revascularization in restoring myocardial function and improving clinical outcomes in ischemic cardiomyopathy patients. An absolute increase in LVEF of 5% or more is generally considered clinically meaningful, which was observed in the OMT + PCI/CABG group. In the representative case, although classified as non-viable, the patient’s LVEF improved, which may be explained by misalignment, partial viability, or response to medical therapy. Despite the significant improvement in LVEF, no significant differences in five-year mortality rates were observed between patients receiving invasive treatments and those receiving only OMT; thus, while revascularization may result in short-term improvements in cardiac function, additional studies on its impact on long-term survival are required, as previously reported by a multicenter trial [24]. Our study provides compelling evidence for the clinical utility of D-SPECT in assessing myocardial viability; however, larger, more diverse patient cohorts and multicenter collaborations are required to validate these findings and optimize the integration of D-SPECT into routine clinical practice.

In recent years, the importance of the quadruple therapy so-called “fantastic four” used as OMT for HF, namely beta-blockers, angiotensin receptor-neprilysin inhibitors, mineralocorticoid receptor antagonists, and sodium-glucose cotransporter-2 inhibitors, has been widely demonstrated [25]. These medications are a cornerstone in the treatment of HF, with strong evidence supporting their effectiveness in lowering morbidity and mortality. The current study was conducted between 2015 and 2017, at a time when beta-blockers and RAS inhibitors were widely used. However, it is worth noting that the full potential of HF therapy may not have been realized during this timeframe. Future research should investigate the impact of optimizing the use of quadruple therapy on patient outcomes.

Our study adds to the existing body of literature by providing direct comparative data on the diagnostic accuracy of D-SPECT and CMR for determining myocardial viability. While previous research demonstrated the efficacy of each method individually, there have been no attempts to directly compare their performance within the same patient cohort. By completing this task, the current study closes a significant knowledge gap and gives clinicians valuable insights into the relative strengths and limitations of both imaging techniques.

We acknowledge some of our study’s limitations, which can help us interpret its findings. First, the sample size of this study is very small, and therefore the comparison between the two groups may lack accuracy. The significant difference in diabetes prevalence between the two groups may have contributed to a potential confounding factor. However, in actual clinical practice, there are very few patients who undergo both D-SPECT and cardiac MRI at the same duration, so in the future, it will be necessary to conduct studies with a larger sample size. Second, the study’s retrospective and observational nature introduces inherent biases that may influence the findings. The prospective analysis also should be needed for the deeper analysis. Third, because our study was conducted at a single institution and includes the interval within 100 days for the two modalities, our findings may be limited in their applicability to other patient populations and clinical settings. Additionally, the Kaplan-Meier survival comparison was exploratory in nature, and the small sample size limited the statistical power to detect meaningful differences between groups. Future prospective studies with larger, more diverse patient cohorts and multicenter collaborations are required to validate the findings reported in this paper and further understand the clinical utility of D-SPECT in myocardial viability assessment. Furthermore, artifacts and false positives in D-SPECT have also been reported [26]; thus, a more thorough comparison with CMR should be focused on in future studies. In particular, false-positive findings may lead to overestimation of myocardial scar burden, potentially resulting in the inappropriate exclusion of patients from revascularization.

Moving forward, several avenues for future research could confirm and extend the current study. First, longitudinal studies with longer follow-up periods are required to evaluate the long-term impact of revascularization on clinical outcomes such as mortality rates and cardiac events. Second, comparative cost-effectiveness studies of D-SPECT and CMR in myocardial viability assessment are expected to provide useful information to healthcare providers and policymakers. This is particularly relevant given the substantial differences in scanner cost, scan time, and institutional accessibility between the two modalities, which may influence decision-making in resource-limited settings. Furthermore, investigating the role of advanced imaging techniques, such as artificial intelligence and machine learning algorithms, may be applied to automated scoring of myocardial viability, standardized image interpretation, and prognostic modeling, enhancing both consistency and clinical value.

In conclusion, our findings provide compelling evidence that D-SPECT is a viable alternative to CMR for assessing myocardial viability in patients with ischemic cardiomyopathy. Due to its comparable accuracy and increased accessibility, D-SPECT has the potential to improve clinical decision-making and outcomes for patients with ischemic heart disease.

## Conclusions

Our findings show that D-SPECT demonstrates comparable accuracy to CMR in assessing myocardial viability, indicating its potential as a reliable alternative imaging modality. These findings support the use of D-SPECT in clinical practice to guide treatment decisions in patients with ischemic cardiomyopathy.
